# Depletion of donor dendritic cells ameliorates immunogenicity of both skin and hind limb transplants

**DOI:** 10.3389/fimmu.2024.1395945

**Published:** 2024-05-10

**Authors:** Muhammad Imtiaz Ashraf, Joerg Mengwasser, Anja Reutzel-Selke, Dietrich Polenz, Kirsten Führer, Steffen Lippert, Peter Tang, Edward Michaelis, Rusan Catar, Johann Pratschke, Christian Witzel, Igor M. Sauer, Stefan G. Tullius, Barbara Kern

**Affiliations:** ^1^ Department of Surgery, Experimental Surgery, Charité – Universitätsmedizin Berlin, Humboldt-Universität zu Berlin and Berlin Institute of Health, Berlin, Germany; ^2^ Department of General, Visceral and Transplant Surgery, Hannover Medical School, Hannover, Germany; ^3^ Department of Pathology, Charité – Universitätsmedizin Berlin, Berlin, Germany; ^4^ Department of Nephrology and Internal Intensive Care Medicine, Charité Universitätsmedizin Berlin, Humboldt-Universität zu Berlin, and Berlin Institute of Healthy, Berlin, Germany; ^5^ Department of Plastic Surgery, Charité – Universitätsmedizin Berlin, Humboldt-Universität zu Berlin and Berlin Institute of Health, Berlin, Germany; ^6^ Division of Transplant Surgery, Brigham and Women’s Hospital, Harvard Medical School, Boston, MA, United States; ^7^ Einstein Berlin Institute of Health Visiting Fellow, Charité – Universitätsmedizin Berlin, Berlin, Germany; ^8^ Berlin Institute of Health at Charité – Universitätsmedizin Berlin, Berlin Institute of Health (BIH) Biomedical Innovation Academy, Berlin Institute of Health (BIH) Charité Clinician Scientist Program, Berlin, Germany

**Keywords:** acute cellular rejection, vascularized composite-tissue allografts (VCA), antigen presenting cells (APCs), conventional dendritic cells (cDCs), allograft immunogenicity, mouse models of skin and hind limb transplantation, Th17 immune response

## Abstract

Acute cellular rejection remains a significant obstacle affecting successful outcomes of organ transplantation including vascularized composite tissue allografts (VCA). Donor antigen presenting cells (APCs), particularly dendritic cells (DCs), orchestrate early alloimmune responses by activating recipient effector T cells. Employing a targeted approach, we investigated the impact of donor-derived conventional DCs (cDCs) and APCs on the immunogenicity of skin and skin-containing VCA grafts, using mouse models of skin and hind limb transplantation. By post-transplantation day 6, skin grafts demonstrated severe rejections, characterized by predominance of recipient CD4 T cells. In contrast, hind limb grafts showed moderate rejection, primarily infiltrated by CD8 T cells. Notably, the skin component exhibited heightened immunogenicity when compared to the entire VCA, evidenced by increased frequencies of pan (CD11b^-^CD11c^+^), mature (CD11b^-^CD11c^+^MHCII^+^) and active (CD11b^-^CD11c^+^CD40^+^) DCs and cDC2 subset (CD11b^+^CD11c^+^ MHCII^+^) in the lymphoid tissues and the blood of skin transplant recipients. While donor depletion of cDC and APC reduced frequencies, maturation and activation of DCs in all analyzed tissues of skin transplant recipients, reduction in DC activities was only observed in the spleen of hind limb recipients. Donor cDC and APC depletion did not impact all lymphocyte compartments but significantly affected CD8 T cells and activated CD4 T in lymph nodes of skin recipients. Moreover, both donor APC and cDC depletion attenuated the Th17 immune response, evident by significantly reduced Th17 (CD4^+^IL-17^+^) cells in the spleen of skin recipients and reduced levels of IL-17E and lymphotoxin-α in the serum samples of both skin and hind limb recipients. In conclusion, our findings underscore the highly immunogenic nature of skin component in VCA. The depletion of donor APCs and cDCs mitigates the immunogenicity of skin grafts while exerting minimal impact on VCA.

## 1 Introduction

Vascularized composite tissue allotransplantation (VCA), the transplantation of multiple tissue types including skin, nerves, muscles, and bones has become an accepted clinical reality: VCA encompasses the reconstructive transplantation of upper and lower extremities in amputees, facial transplantation for craniofacial tissue defects and the transplantation of reproductive organs (1–4). VCA recipients experience exceptionally high rates of acute rejection episodes, affecting approximately 85% of patients within first year, with figures reaching as high as 100% in some centers (5, 6). The elevated incidence of acute cellular rejection (ACR) in VCA can be attributed to the access and ease of obtaining skin biopsies (7), but has been primarily linked to the augmented immunogenicity of its skin component (5). ACR of the skin component in VCA responds in general quite well to the treatment, with only few graft losses. More frequent acute rejection rates, at the same time, may increase the risk of graft loss with increased rates of chronic rejection and vasculopathy. A detailed understanding of VCA immunology is thus a prerequisite to improve the graft management through the development of targeted therapeutic strategies (5, 7).

Current immunosuppression protocols in VCA are primarily based on standard protocols used in solid organ transplantation (SOT). These protocols typically involve an antibody-based induction therapy with polyclonal antithymocyte globulin (ATG) or monoclonal antibodies (mAb) including alemtuzumab or basiliximab, followed by maintenance immunosuppressive regimens usually consisting of tacrolimus, mycophenolate mofetil, and prednisolone (8). While these protocols have shown partial effectiveness in VCA, the lifelong use of immunosuppressive regimens comes with considerable risks and unwanted effects, including infections, metabolic disorders, and malignancies. These risks must be weighed against the life-enhancing, though only rarely life-saving nature of VCA therapy (9, 10). To ensure long-term graft acceptance through specifically tailored immunosuppression protocols for VCA patients, a comprehensive understanding of the underlying mechanisms during early and late immune responses to VCA is imperative.

The initial alloimmune response against the graft is triggered by donor-derived antigen presenting cells (APCs), primarily dendritic cells (DCs) (11–14). Donor DCs activate recipient alloreactive T cells either by directly presenting intact antigens after migrating to the draining lymph nodes or through transfer of intact donor major histocompatibility (MHC)-antigen complexes to recipient DCs. Immunogenic and regulatory role of graft-derived DCs has been documented in skin and solid organ transplantations (11–16). However, it is still under discussion if VCA-derived DCs predominantly facilitate graft acceptance or trigger immune activation that leads to rejection.

Here, we aimed at distinguishing recipient’s alloimmune response between isolated skin and the entire VCA grafts utilizing mouse models of hind limb and skin transplantation. Moreover, we investigated the influence of APCs, particularly cDCs, on initiating and/or modulating the alloimmune response in VCA compared to skin transplants. Our findings underscore a more potent immunogenicity of the skin component relative to the entire VCA and emphasize that pre-implant depletion of donor DCs and APCs ameliorates the immunogenicity of murine skin and hind limb transplants through a modulation of Th17 responses in recipient animals.

## 2 Materials and methods

### 2.1 Animals

Female C57BL/6J (H-2k^b^, donor) and DBA/2J (H-2k^d^, recipient) mice were purchased from Charles River Laboratories (Sulzfeld, Germany) or obtained from the central animal facility (Forschungseinrichtungen für Experimentelle Medizin, FEM) of the Charité – Universitätsmedizin Berlin, Germany. Breeding pairs of B6(Cg)-*Zbtb46^tm1(HBEGF)Mnz^*/J (B6 zDC-DTR, Jax Mice, stock number: 019506) were purchased from Jackson Laboratory (Bar Harbor, ME, USA) and bred in the FEM at Charité. In zDC-DTR knock-in mice, human diphtheria toxin (DT) receptor (DTR) is integrated into the promoter region of cDC-specific zinc finger transcription factor (zDC). Treatment with diphtheria toxin ensures targeted depletion of cDCc in these mice (17). Mice, weighing 20-24 g, were used for all the experiments and had *ad libitum* access to food and water. Animals received human care in compliance with the ‘Principles of Laboratory Animal Care’ prepared by the National Academy of Sciences and published by the National Institutes of Health (NIH Publication No. 86-23, revised 1985). The State Office for Health and Social Affairs (Landesamt für Gesundheit und Soziales), Berlin approved and supervised all animal experiments (approval number: G0300/17).

### 2.2 *In vivo* treatment

For cDC depletion zDC-DTR knock-in mice received an intraperitoneal (i.p.) injection of 10 ng of DT (*Sigma-Aldrich*, St. Louis, MO, USA) per gram of body weight 15 hours before transplantation. Transient depletion of all antigen presenting cells (APCs) was achieved by injecting 100 µl clodronate liposomes intravenously (i.v.) into C57BL/6J on day -8, -5 and -1 before transplantation, as previously described (18). All control mice received the same amount of PBS liposomes (*Liposoma B.V.*, The Netherlands) using the same injection schedule.

### 2.3 Orthotopic hind limb transplantation

Hind limb transplantations in the mouse model were performed under inhalation anesthesia with isoflurane (2%) as previously described (19). Briefly, the left hind leg of the donor mouse was prepared by shaving and disinfecting the skin of the thigh region, followed by a circumferential cut in the center of the femur. The femoral artery and vein were isolated and dissected to ensure sufficient length for subsequent vascular anastomoses. The remaining thigh muscle groups were then severed, followed by a transverse femur osteotomy with a sharp scalpel. The grafts were perfused via femoral artery with 2 ml of ice-cold histidine-tryptophan-ketoglutarate (HTK) solution (*Cardiolink*, Barcelona, Spain). Polyamide cuffs were placed over the femoral vessels, followed by cold storage of the graft in ice cold HTK solution for approximately 1 hour until transplantation.

In recipient mice, the equilateral hind leg was removed congruently after clamping the femoral artery and vein proximal to the circumferential cut. The graft was first orthotopically connected to the recipient via femoral osteosynthesis using an intramedullary rod made from a 22-gauge blunt cannula. The femoral vein and artery were then anastomosed between donor and recipient with a cuff technique. Finally, the muscles were adapted in layers, followed by anastomosis of the skin with 6-0 *Vicryl™* (Ethicon/Johnson&Johnson Medical GmbH, Norderstedt, Germany) suture. The animal was kept on a warming pad throughout the operation. For post-operative analgesia, animals received 0.05 mg/Kg BW of Buprenorphine (*Temgesic^®^*, *Indivior Europe Limited Dublin*, Ireland) and 5 mg/Kg BW of Carprofen (*Rimadyl^®^*, *Pfizer*, Berlin, Germany).

### 2.4 Skin transplantation

Skin transplants in the mouse were performed based on established anesthetic and analgesic protocols, described for hind limb transplantation. Full thickness skin grafts were procured from donor’s dorsum with blunt dissection at the level of the areolar connective tissue under sterile conditions and prepared prior to transplantation via separation of the epidermis from the remaining tissue. The skin grafts were kept on ice for approximately 1 hour prior to transplantation, aligning with unavoidable cold ischemia time incurred during the transplantation of murine hind limb grafts. A transplant bed of approximately 10 x 10 mm was prepared on the dorsal trunk of the recipient mouse by removing a narrow strip of epidermis including hair follicles without injuring the panniculus carnosus. The donor skin patch, precisely adopted to the wound edges, was placed on the transplant bed and fixed with intact 6-0 *Vicryl™* sutures.

### 2.5 Immune cell isolation

To isolate graft mononuclear cells (MNCs), tissues procured from transplanted skin and VCA grafts were cut into smaller pieces and digested in Hanks Balanced Salt Solution (HBSS), supplemented with 1.5 mg/ml Collagenase P and 0.4 mg/ml DNAse I (both from *Roche*, Grenzach-Wyhlen, Germany) for 45 minutes at 37° C. Peripheral blood mononuclear cells (PBMCs) were isolated from blood samples, taken via cardiac puncture. MNCs from lymph nodes and spleen were isolated by gently grinding the lymph node and spleen pieces through a 40 µm strainer with the help of a syringe plunger. Erythrocytes were eliminated from the PBMCs and splenocyte samples using ACK Lysis buffer. All isolated cells were collected in MACS buffer (PBS, 0.5% BSA, 0.5 mM EDTA) and maintained on ice until antibody labelling for flow cytometry.

### 2.6 Flow cytometry

For flow cytometry analysis, 2x10^5^ isolated cells per sample were incubated in 100 µl MACS buffer for 20 minutes in the dark with different sets of antibodies, as specified in **Supplementary Table 1**. For intracellular labelling, cells were fixed and permeabilized using the *Cyto-Fast*™ Fix/Perm kit from *BioLegend* ^®^ San Diego, CA, USA, according to the manufacturer’s protocol prior to incubation with antibodies. 4′, 6-diamidino-2-phenylindole (DAPI) was added just before the analysis for live/dead cell discrimination. All flow cytometry investigations were performed on a *BD LSRFortessa*™ X-20 device (BD Biosciences, Heidelberg, Germany) with *FACS Diva* software, and the data sets were analyzed using the *FlowJo^®^* V10 software (BD, Ashland, OR USA).

### 2.7 Multiplex cytokine analysis

Analysis of serum cytokines was performed using multiplex immunoassay. For isolation of serum, the blood samples taken from transplanted mice were first kept on ice for 1 hour to allow clotting. Subsequently, samples were centrifuged for 10 minutes at 3500 g to separate serum from cellular components. Serum samples were stored at -80° C until the analysis was performed on all samples together. Cytokine expression was analyzed using the *Milliplex^®^* Mouse Th17 magnetic bead panel kit (Cat#: MT17MAG47K-PX25, Merck, Darmstadt, Germany) according to the manufacturer’s protocol using a *Luminex MagPix^®^* instrument and the *xMAP^®^* software (Luminex Co, Hertogenbosch, The Netherlands).

### 2.8 Cytokine qPCR

mRNA from fresh-frozen samples of transplanted skin and hind limb tissues was isolated using the Qiagen EasyRNA^®^ kit and transcribed into cDNA using the High-Capacity cDNA Reverse Transcriptase kit (*Thermo Fisher Scientific* ^®^). Real-time PCR was performed with *Thermo Scientific* ^®^ Applied Biosystems TaqMan Gene expression master mix and TaqMan Probes primer annotated in **Supplementary Table 2** using an Applied Biosystems StepOnePlus device.

### 2.9 Macroscopic evaluation of graft rejection

Recipient animals were monitored daily for gross appearance of skin and hind limb grafts and documented via photographs. Graft perfusion was evaluated via capillary refill test of toes or skin grafts within 2-3 seconds. Clinical signs of rejection were classified as previously described: Grade 0 – no signs of rejection; Grade I – erythema of the graft; Grade II – erythema and edema; Grade III epidermolysis of the graft; Grade IV – mummification of the graft (20). Hind limb recipients with graft failure due to vascular complications were excluded from the analysis.

### 2.10 Histopathology

Tissue samples of the skin and VCA transplants were fixed in 4% paraformaldehyde solution (PFA) for up to 24 hours, followed by processing for paraffin embedding using a *Thermo Scientific* ^®^ Excelsior ES Tissue Processor. Sections of 4 µm thickness were prepared, deparaffinized with Xylene and a descending ethanol series, and stained with Mayer’s Hematoxylin and Eosin (both Morphisto GmbH, Frankfurt a.M., Germany, cat# 10231 and 11503) as described before (19). Sections were blinded and histopathological analysis was performed according to the respective Banff criteria for skin and VCA transplants (21).

### 2.11 Statistics

Statistical analysis were performed using *GraphPad Prism* (version 8.4.3, GraphPad Software, La Jolla, CA). The D’Agostino & Pearson omnibus normality test was used to assess normal data distribution. Data were expressed as the mean ± standard error of the mean (SEM). Ordinary One-Way ANOVA (Analysis of Variance) or non-parametric Kruskal-Wallis test followed by appropriate multiple comparison tests were performed to compare more than two groups. Two-Way ANOVA was applied in case of two categorical variables (type of transplant, pretreatment group) followed by Tukey’s or Sidak’s multiple comparison tests, respectively. A *p*-value of less than 0.05 was considered significant.

## 3 Results

### 3.1 Targeted depletion of professional APCs and cDCs in the donor mice

To elucidate the impact of donor-derived cDCs on the immunogenicity of composite tissue and skin allotransplantation, we selectively depleted cDCs in zDC-DTR knock-in mice and all professional APCs in C57Bl/6 donor mice. To ensure toxicity safety, we first titrated DT in zDC-DTR mice, determining 10 ng/g of body weight as an effective dose, reducing frequencies of DCs in peripheral blood and spleen of the treated mice with no signs of toxicity within 15 hours (**Supplementary Figure 1A**). This dose was consistently applied for subsequent cDC depletion experiments. Similarly, intravenous administration of clodronate liposomes (100 µl) to donor C57BL/6J mice on days -8, -5 and -1 prior to transplantation significantly reduced the number of all APCs, including macrophages and DCs in peripheral blood and spleen of the treated mice (**Supplementary Figures 1A, B**) (18). Importantly, the analysis of donor-specific DCs (H2Kb^+^CD11c^+^) in the skin and hind limb grafts at POD 6 showed significantly reduced numbers of cDCs in both zDC (DT treated zDC-DTR mice) and CL (clodronate treated C57BL/6J mice) groups, compared to the WT (PBS treated C57BL/6J mice) group (**Supplementary Figure 1C**).

### 3.2 Depletion of donor cDCs and APCs influences rejection of murine skin and hind limb allografts

**Figure 1:**
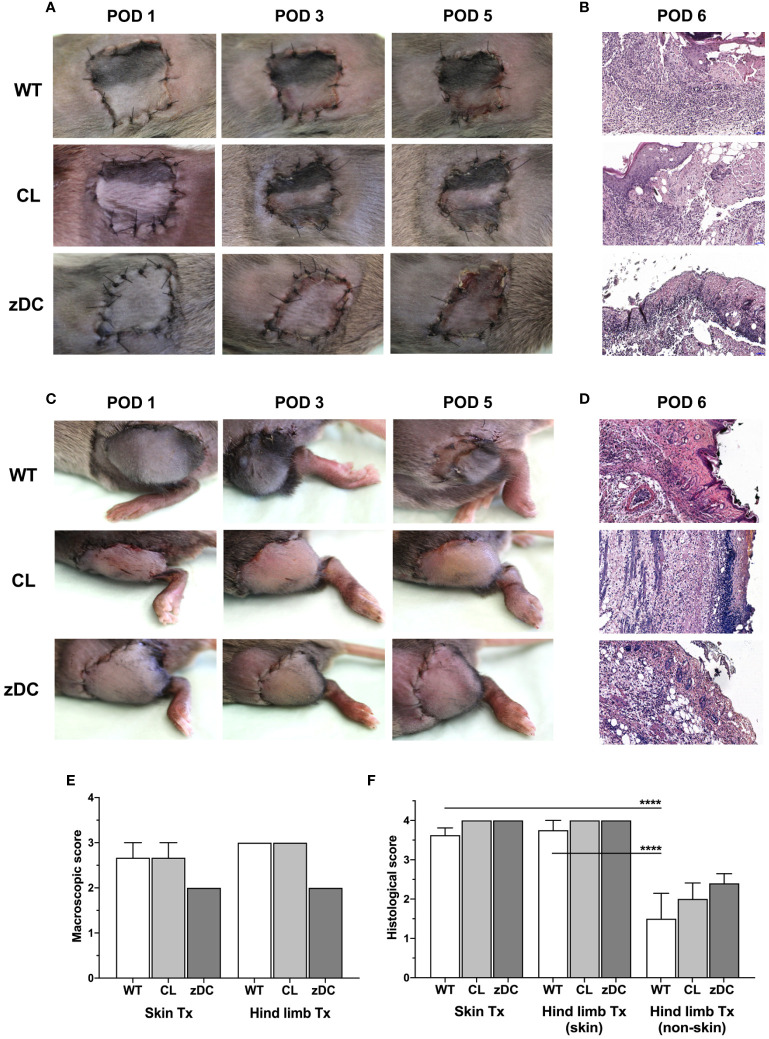
Effect of donor APC and cDC depletion on mouse hind limb and skin allografts. Hind limb and skin grafts of untreated control (WT) and APC depleted (CL; clodronate treated) C57Bl/6 mice and cDC depleted (zDC; DT treated zDC-DTR) mice were transplanted in DBA/2 mice. The grafts were macroscopically evaluated for acute rejection as previously defined (20). Representative images of the hind limb **(A)** and skin **(C)** transplants on post-operative day (POD) 1, 3 and 5 are shown. Representative images of H&E-stained sections of the hind limb **(B)** and skin **(D)** allografts of untreated (WT), APC depleted (CL) and cDC depleted (zDC) donor mice, harvested on POD 6 are shown (20x magnification) (n=5). The rejection scores of the macroscopic evaluation **(E)** (n=3) and Banff classification **(F)** (n=8 skin Tx, n=5 hind limb Tx) of the hind limb and skin allografts are presented by the bar graphs. ****p<0.0001.

To evaluate the impact of donor cDC on skin and hind limb transplant outcomes, allografts from cDC and APC depleted mice were transplanted into DBA/2J WT mice. Of note, immunosuppression was not used to study an unbiased effect of DCs on acute rejections. Both skin and hind limb allografts were macroscopically examined daily and scored based on the clinical signs of acute rejection, as previously described (20). Skin and hind limb transplants exhibited progressive signs of acute rejection by day 5 (**Figures 1A, C**). Compared to the WT and APC depletion groups, cDC depleted grafts exhibited ameliorated, albeit not statistically different allograft rejection scores (**Figures 1E**). Histopathological evaluation by post-operative day (POD) 6 revealed severe rejection of hind limb and skin transplants, with highest possible Banff scores (4) (**Figure 1F**). The Banff criteria for skin-containing composite tissue allotransplantation are restricted to the skin component of the transplant. The analysis of the underlying non-skin VCA (muscular and connective tissue layers) demonstrated lower rejection scores (<2), which is known to show reduced immunogenicity relative to the skin component (22). Nonetheless, no significant differences were observed between untreated WT groups and those with donor APC or cDC depletion in both skin and hind limb transplants.

### 3.3 Depletion of donor cDCs impacts recipient DC activity

**Figure 2:**
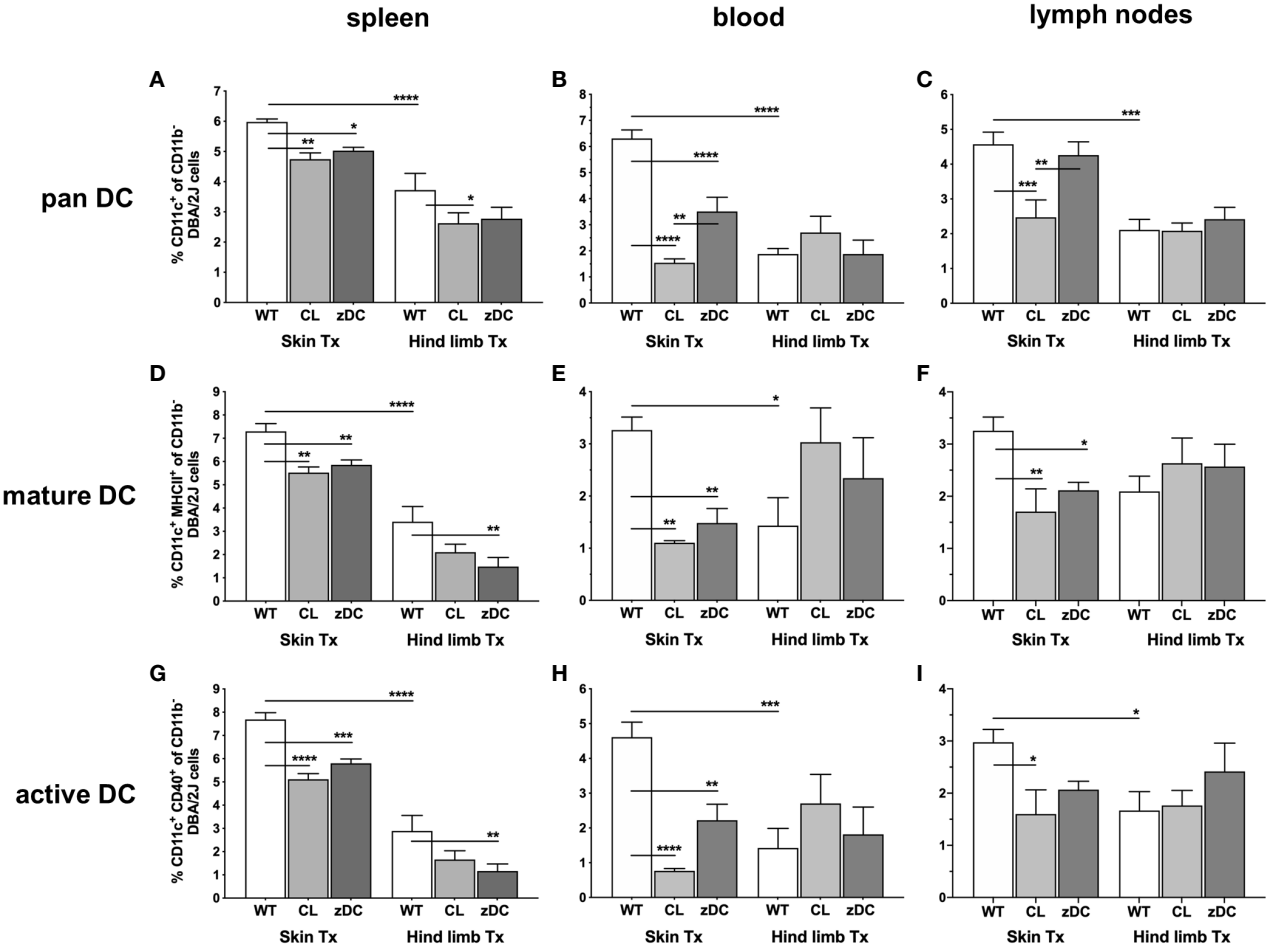
Donor APC and cDC depletion variably impacts abundance, maturation, and activation of recipient DCs, following murine skin and hind limb transplantation. Immune cells were isolated from blood and lymphoid tissues (spleen and lymph nodes) of the recipients (DBA/2 mice) of skin and hind limb grafts-derived from untreated (WT), APC depleted (CL; clodronate treated) and cDC depleted (zDC; DT treated zDC-DTR) donor mice at POD 6 and analyzed by flow cytometry. The bar graphs indicate frequencies of pan (CD11b^-^CD11c^+^) **(A–C)**, mature (CD11b^-^CD11c^+^MHCII^+^) **(D–F)** and active (CD11b^-^CD11c^+^CD40^+^) **(G–I)** DCs gated on recipient H2Kd^+^ CD45^+^ cells. The gating strategies are described in **Supplementary Figure 2**. All values are given in % of the described population on the ordinate/”y-axis”. (n=8 skin Tx, n=5 hind limb Tx). *p<0.05, **p<0.01, ***p<0.001, ****p<0.0001.

To understand the systemic impact of donor cDC depletion on skin and hind limb graft immunogenicity, we first characterized recipient DCs in blood and secondary lymphoid compartments (spleen, lymph nodes) of recipient mice procured on POD 6. The frequency of pan DCs (CD11b^-^CD11c^+^) were markedly higher in all analyzed tissues of recipients of skin WT grafts compared to recipients of hind limb (HL) WT grafts (**Figures 2A–C**). Notably, DC counts in mice receiving a skin transplant from APC depleted clodronate-treated donors (CL) were consistently reduced in all the compartments. Similarly, DT-induced depletion of cDC in zDC-DTR donor mice (zDC) significantly reduced DC counts in the spleen and blood of skin graft recipients. However, depletion of APCs or cDCs in the donor had minimal effects on the already low DC numbers in recipients of hind limb, except on splenic DCs of clodronate treated group (CL). Furthermore, the frequency of mature (CD11b^-^CD11c^+^MHCII^+^) and active (CD11b^-^CD11c^+^CD40^+^) DCs was higher in almost all the analyzed tissues of the recipients of skin WT grafts compared to recipients of hind limb WT grafts (**Figures 2D–I**). Depletion of either donor APCs or cDCs resulted in a significant decrease of mature and active DCs in the spleen, blood, and lymph nodes of skin graft recipients. However, this reduction was exclusively observed in the spleen of recipients who received hind limb from cDC depleted donors (**Figures 2D, G**).

**Figure 3:**
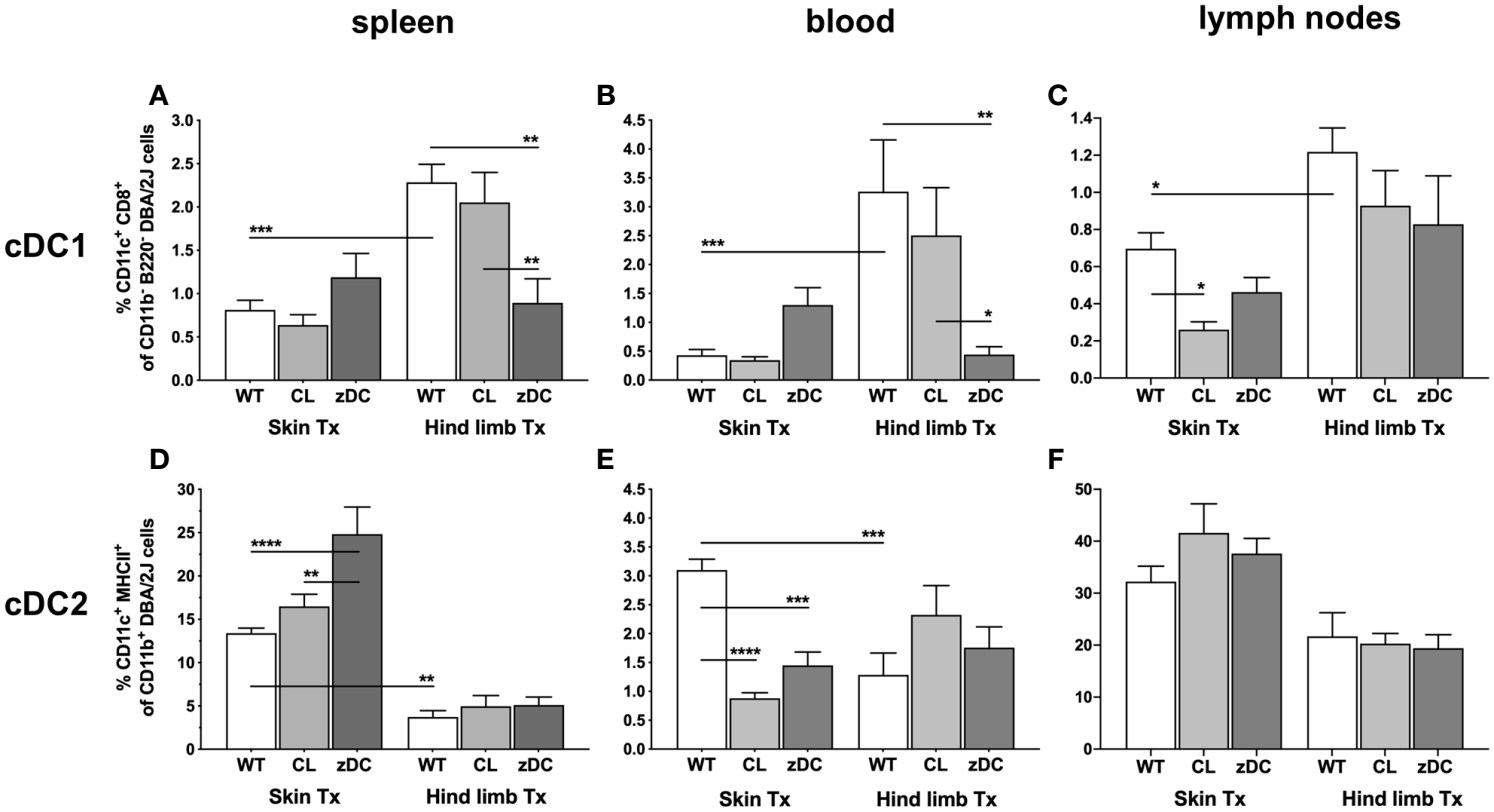
Donor APC and cDC depletion demonstrate diverse impacts on two major subsets of DCs, following murine skin and hind limb transplantation. Immune cells were isolated from blood and lymphoid tissues (spleen and lymph nodes) of the recipients (DBA/2 mice) of skin and hind limb grafts-derived from untreated (WT), APC depleted (CL; clodronate treated) and cDC depleted (zDC; DT treated zDC-DTR) donor mice at POD 6 and analyzed by flow cytometry. The bar graphs indicate frequencies of two major subsets of cDCs, cDC1 (CD11b^-^B220^-^CD11c^+^CD8^+^) **(A–C)** and cDC2 (CD11b^+^CD11c^+^MHCII^+^) **(D–F)** in the specified organs. The cDCs were gated on recipient-specific H2Kd^+^ CD45^+^ cells. The gating strategies are described in **Supplementary Figure 3**. All values are given in % of the described population on the ordinate/”y-axis”. (n=8 skin Tx, n=5 hind limb Tx). *p<0.05, **p<0.01, ***p<0.001, ****p<0.0001.

Focusing on the subtypes of cDCs, we observed that numbers of lymphatic tissue resident type 1 cells (cDC1 subset: CD11c^+^CD8^+^ of CD11b^-^/B220^-^) were significantly higher in all analyzed tissues of hind limb WT graft recipients compared to recipients of WT skin grafts. Only spleen and blood in cDC depleted hind limb group (zDC) demonstrated reduced cDC1 numbers (**Figures 3A–C**). Conversely, numbers of type 2 cDCs (CD11c^+^MHCII^+^ of CD11b^+^) were significantly elevated in recipient’s spleen and blood following WT skin transplantation compared to WT hind limb (**Figures 3D–F**). Interestingly, depletion of donor APC (CL) or cDC (zDC) resulted in decreased cDC2 counts in the blood of skin graft recipients, whereas augmented cDC2 numbers were observed in the spleen of skin graft recipients of zDC animals.

### 3.4 Donor cDC and APC depletion distinctly influence the T cell response following skin and hind limb transplantation

**Figure 4:**
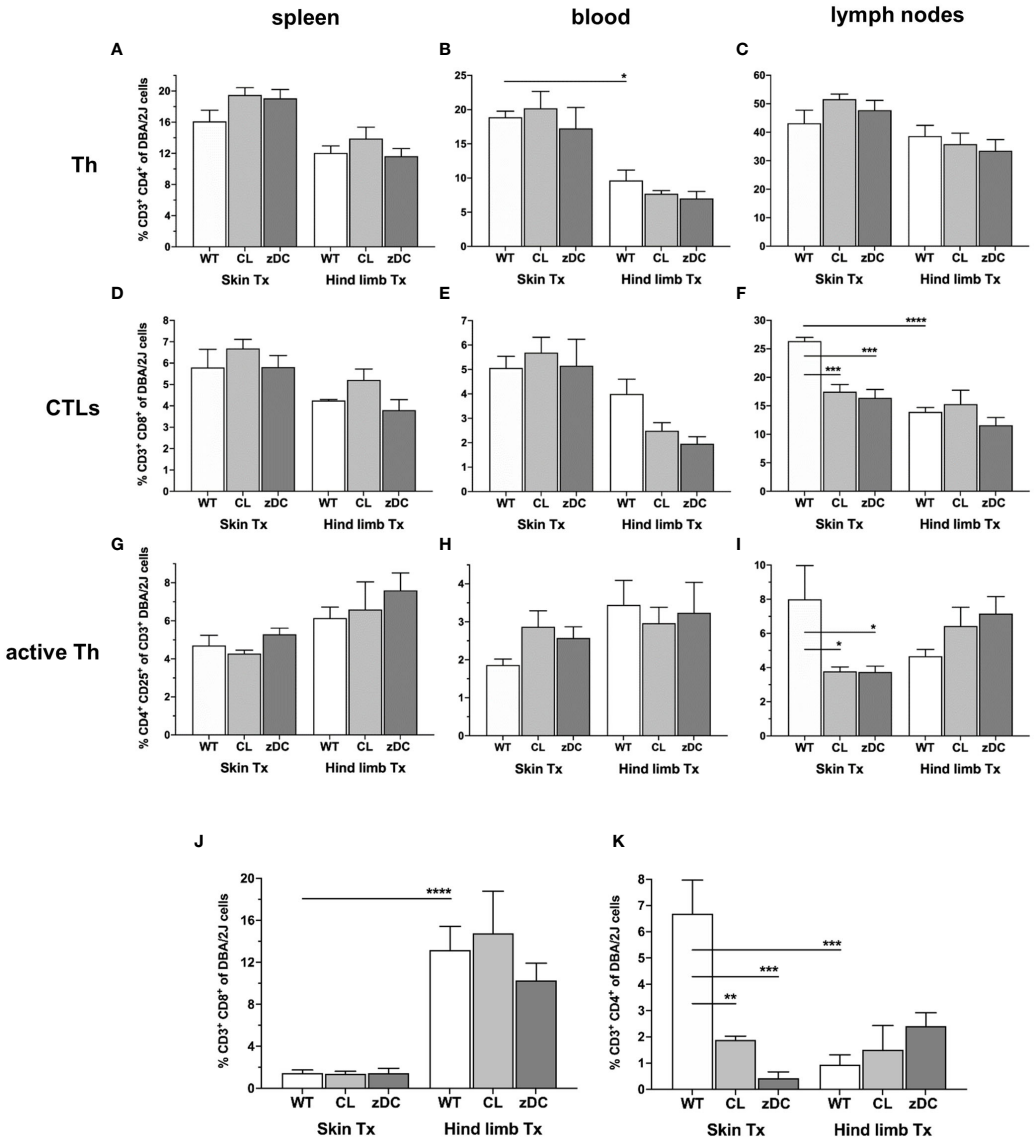
Effect of donor APC and cDC depletion on recipient T lymphocytes, following murine skin and hind limb transplantation. Immune cells were isolated from blood, lymphoid tissues (spleen and lymph nodes) and the allografts of the recipients (DBA/2 mice) of skin and hind limb grafts-derived from untreated (WT), APC depleted (CL; clodronate treated) and cDC depleted (zDC; DT treated zDC-DTR) donor mice at POD 6 and analyzed by flow cytometry. The bar graphs indicate frequencies of two major subsets of T lymphocytes, Th (CD3^+^CD4^+^) **(A–C)** and CTLs (CD3^+^CD8^+^) **(D–F)** and active Th cells (CD3^+^CD4^+^CD25^+^) **(G–I)** isolated from spleen, blood and lymph nodes of the recipients. **(J, K)** The bar graphs indicate frequencies of recipient CD8^+^ **(J)** and CD4^+^ **(K)** T cells infiltrated in the skin and hind limb grafts of the recipients at POD 6 in untreated (WT), APC depleted (CL) and cDC depleted (zDC) groups. The lymphocytes were gated on recipient-specific H2Kd^+^ CD45^+^ cells. The gating strategies are described in **Supplementary Figure 2**. All values are given in % of the described population on the ordinate/”y-axis”. (n=8 skin Tx, n=5 hind limb Tx). *p<0.05, **p<0.01, ***p<0.001, ****p<0.0001.

To delineate the effector response subsequent to dendritic cell-initiated immune responses, we next analyzed T cell responses in recipients of both skin and hind limb transplants. Notably, numbers of CD3^+^CD4^+^ Th cells were significantly higher in the blood of WT skin graft recipients when compared to WT hind limb recipients; cDC or APC depletion did not impact this outcome; neither did we observe differences in splenic and lymph nodes samples of either WT or DC depleted groups (**Figures 4A–C**).

Likewise, splenic and peripheral blood CD3^+^CD8^+^ T cell counts did not differ significantly, neither between the two transplant models nor between the wild type and DC depletion groups. However, the frequency of CD3^+^CD8^+^ T cells was significantly higher in lymph nodes of mice receiving a skin WT grafts compared to those receiving hind limb WT grafts (**Figures 4D–F**). Interestingly, both the depletion of donor cDCs (zDC) and APCs (CL) resulted in a significant reduction of lymph nodes CD3^+^CD8^+^ T cells in skin transplant recipients.

Numbers of activated CD4^+^CD25^+^ T cells did not show differences in spleen and blood samples of the skin and hind limb transplant recipients. In contrast, in the lymph nodes of skin graft recipients, the depletion of donor cDCs (zDC) and APCs (CL) resulted in a significant reduction of CD4^+^CD25^+^ T cells (**Figures 4G–I**).To elucidate the effector responses within the skin and hind limb allografts, we analyzed the composition of intra-graft T lymphocyte. Notably, grafts were consistently infiltrated with recipient lymphocytes with hind limb grafts predominantly showing CD3^+^CD8^+^ T cell infiltration and skin grafts showing CD3^+^CD4^+^ T cell dominance (**Figures 4J, K**). Depletion of donor cDC (zDC) or APC (CL) had no impact on the frequencies of CD3^+^CD8^+^ T cells in both skin and hind limb grafts (**Figure 4J**). However, while the number of CD3^+^CD4^+^ T cells remained unaffected in hind limb grafts of both donor cDC (zDC) and APC (CL) depleted groups, frequencies of CD3^+^CD4^+^ T cells were significantly reduced in the skin grafts of both depleted groups compared to the WT group (**Figure 4K**). This suggests an influence of donor cDC and APC depletion on the Th (CD3^+^CD4^+^ T cells) mediated effector response within the skin grafts.

### 3.5 Effect of donor APC and cDC depletion on Th17 response in skin and hind limb transplantation

**Figure 5:**
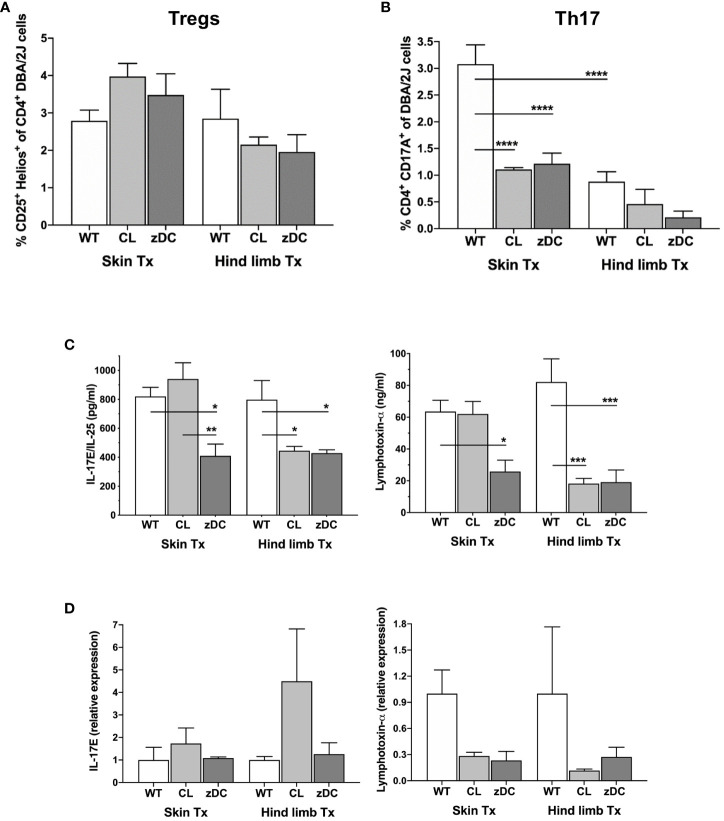
Effect of donor APC and DC depletion on Th17 mediated alloimmune response. Splenocytes were isolated from the recipients (DBA/2 mice) of skin and hind limb grafts-derived from untreated (WT), APC depleted (CL; clodronate treated) and cDC depleted (zDC; DT treated zDC-DTR) donor mice at POD 6 and analyzed for the expression of IL-17 and Hellios in CD4^+^ T cells using flow cytometry. The bar graphs indicate frequencies of Tregs (CD4^+^Hellios^+^) **(A)** and Th17 (CD4^+^IL17^+^) **(B)** cells, respectively. The CD3^+^ T cells were gated on CD3^+^CD45^+^ cells. The gating strategies are described in **Supplementary Figure 4**. All values are given in % of the described population on the ordinate/”y-axis”. Serum was isolated from the blood samples of the skin and hind limb recipients at POD 6 and subjected to multiplex analysis for the levels of Th17-related cytokines IL17E/IL-25 and lymphotoxin-α. The representative bar graphs are shown **(C)**. **(D)** Quantitative RT-PCR analysis of IL17/IL-25 and lymphotoxin-α expression in the skin and hind limb grafts at POD 6. (n=8 skin Tx, n=5 hind limb Tx). *p<0.05, **p<0.01, ***p<0.001, ****p<0.0001.

To differentiate between Th mediate proinflammatory and regulatory responses we next analyzed the responses of Th17 (CD4^+^IL-17^+^) and Tregs (CD4^+^Helios^+^) Th cells, respectively. While there were no differences in regulatory T cell numbers between the two transplant models or between the WT and depletion groups, Th17 cell counts were significantly elevated in spleen samples of WT skin recipients, compared to WT hind limb transplanted mice (**Figures 5A, B**). Interestingly, in the skin transplant model, Th17 cells were strongly reduced in both APC and cDC depletion groups compared to the respective WT groups, suggesting an effect of both donor APC and cDC depletion on the Th17 immunity of skin recipients.

Multiplex analysis of various proinflammatory and regulatory Th cytokines in the serum samples of the skin and hind limb recipients at POD 6 revealed that most cytokine levels remained unaffected by donor cDC and APC depletion (**Supplementary Figure 5**). Donor cDC depletion in skin transplant recipients and both cDC and APC depletion in hind limb transplant recipients reduced the serum levels of Th17-related cytokines, namely IL-17E and lymphotoxin-α, indicating an attenuation of systemic Th17 response (**Figure 5C**). Likewise, intragraft gene expression of lymphotoxin-α was reduced, albeit not statistically significant, in both APC and cDC depletion groups of skin and hind limb transplants (**Figure 5D**). Interestingly, while the expression of proinflammatory cytokines IFN-γ and IL-6 was reduced in the skin grafts of the cDC depletion group, the expression of TNF-α was increased (**Supplementary Figure 6**).

## 4 Discussion

Compared to solid organs, VCA transplants have a higher incidence of acute rejections, primarily attributed to the presumed highly immunogenic nature of its skin component. To distinguish the rejection phenotype and the nature of immune responses against skin versus the whole vascularized composite allograft, we performed a simultaneous analysis of isolated skin and hind limb grafts in the mouse model. The isolated skin grafts and the skin component of VCA demonstrated severe rejection, based on Banff score. However, the underlying muscular and connective tissues of the VCA were protected (**Figure 1**). Preclinical studies in the rat (23) and swine models (24), as well as clinical reports (25) have consistently shown severe rejections of the skin component of VCA compared to milder rejections of the underlying tissues. The recipient immune systems’ selective targeting of the skin component in VCA can be attributed to the abundance of skin-resident APCs, including DCs and Langerhans cells (10, 26). Additionally, skin serves as a reservoir for a large number of effector memory T cells generated in response to the continuous exposure of skin to foreign antigens (10, 27). The presence of both APCs and effector memory T cells within the skin intensifies the recipients’ immune response against the skin component of VCA. However, conversely, severe rejection of the non-skin components in the absence of clinically detectable skin rejection has also been reported (28, 29). Particularly, the mucosa of the skin in case of facial transplantation seems to show more distinct patterns of acute rejections than the skin component (30). Apparently, the nature and degree of rejection may substantially vary depending on the type of VCA and the tissue composition underlying the skin.

Consistent with the variable rejection of skin and hind limb grafts, our study revealed a notable distinction in the alloimmune response to isolated skin versus skin-containing VCA transplants. Specifically, in our experiments skin grafts were predominantly infiltrated by recipient CD3^+^CD4^+^ T cells, while hind limb grafts were characterized by CD3^+^CD8^+^ T cell infiltrates (**Figures 4J, K**). These observations indicate a favored CD4 T cell-driven adaptive immune response during acute rejection in skin-only allogeneic transplantation and a distinct CD8 T cell-driven rejection process in skin-containing VCA. The preference of T cell subsets in acute rejection has been described for certain organs like skin and heart (31–33). The observed graft-specific responses of T cell subsets may be linked to the elevated cDC1 and cDC2 numbers in secondary lymphoid tissues of VCA and skin transplanted animals, respectively. This is in line with the previous findings that highlight the predominant role of spleen and lymph node resident cDC1 in activating CD8^+^ T cells, while the primary function of the resident cDC2 is to activate CD4^+^ T cells (34). Furthermore, in line with the lower degree of overall rejection of the VCA, we detected lower numbers of pan, mature and active DCs, cDC2 subset and, simultaneously, reduced frequencies of CD4 and CD8 T cells in VCA recipients compared to skin allograft recipients. These data suggest a dampened indirect and semi-direct activation of effector T cells, indicating a reduced systemic adaptive immune response during acute rejection in VCA compared to the skin transplantation model. In addition, we observed reduced Th17 cells in spleen samples of hind limb transplanted mice, compared to skin (**Figure 5B**), suggesting an overall lower antigenicity of the VCA compared to the isolated skin graft, despite the much higher mass and skin area of the VCA graft. Larger allografts typically contain a greater number of antigens, which can elicit a more robust immune response from the recipient’s immune system, leading to more severe rejection episodes. Skin, being the body’s outer barrier, is constantly exposed to a plethora of environmental antigens, including pathogens, allergens, and foreign substances. This continuous exposure results in a higher antigen load in the skin compared to deeper tissues like muscle. Moreover, the skin hosts a dense population of specialized APCs, such as Langerhans cells in the epidermis and dendritic cells in the dermis, which contribute to its heightened immunogenicity compared to deeper muscle tissue (10, 22, 35). We anticipate that the lower overall immune response against the VCA graft might be due to the relative larger proportion of the less immunogenic non-skin component of the VCA, compensating the collective immune response. The slower rate of rejection in VCA could also be due to T cell exhaustion, attributed to the larger size of the highly immunogenic skin component of VCA compared to smaller size of isolated skin grafts. This concept is supported by a recent findings from Zou et al., which demonstrate that T cell exhaustion develops in the presence of higher antigen load and can promote transplant acceptance (36).

Skin grafts were not reconnected to the blood supply during transplantation. Although skin grafts have been placed on a very well vascularized bed, a certain degree of extended ischemia cannot be ruled out. Ischemia and reperfusion injury (IRI) may also occur subsequent to neovascularization that may initiate a cascade of events leading to enhanced proinflammatory responses to the allografts (37, 38). Therefore, the variations in immune responses observed between the non-vascularized skin grafts and the vascularized hind limb grafts could potentially be attributed to the inevitable period of prolonged ischemia experienced by the skin grafts.

Recognizing the pivotal role of graft accompanying APCs in defining immunogenicity, especially during early alloimmune responses, we selectively depleted APCs and cDCs in donor mice, followed by skin and hind limb transplantation into the fully MHC mismatched recipient mice. While there was a slight improvement with donor cDC depletion in the macroscopically evaluated hallmarks of acute rejection for both skin and VCA grafts, the effect was not statistically significant (**Figures 1A, C, E**). We posit that the sole depletion of donor cDCs or APCs might have been insufficient to yield a substantial protective effect in the stringent allograft rejection models with complete MHC mismatch. Additionally, we had deliberately avoided the use of immunosuppression to elude any side effect of immunosuppression on DC and T cell responses. Interestingly, mouse skin grafts depleted of cDCs and transplanted into minor histocompatibility mismatched recipients exhibited prolonged survival, suggesting a protective effect in a situation of a mild alloimmunity (39). It is noteworthy that even minor mismatches were sufficient to induce rejection of the mouse skin grafts, underscoring the robust nature of the skin graft rejection (39). Moreover, in studies involving major histocompatibility mismatched recipients, prolonged survival of murine hearts from DC depleted donors was observed (18, 40), but the same could not be replicated for skin grafts (40). Apparently, the role of donor-derived DCs in rejection appears to be organ-specific and contingent on the histocompatibility match between the donor and receipt.

After transplantation, donor DCs undergo stimulation and migrate to the nearby lymph nodes, where they prime effector T cells for alloantigen-specific immune responses. This activity of donor DCs is most impactful during the initial stages of acute cellular rejection, as these cells are frequently targeted and eliminated by recipient cytotoxic T and NK cells (41, 42). Similarly, we rarely observed donor DCs in blood or secondary lymphoid organs of the recipients at POD 6, neither in the cDC and APC depleted nor in the WT groups (**Supplementary Figures 7A**, **8**). However, a small proportion of cells were double positive, expressing both donor (H2Kb)- and recipient (H2Kd)-specific MHC class I molecules. Since all the double positive cells are mature (MHCII^+^) and active (CD40^+^) (**Supplementary Figure 7B**), we anticipate that these are recipient cells cross-dressed with donor MHC-antigen complexes. However, further studies should confirm the precise phenotype of these cells, and thus the nature of antigen presentation. Nevertheless, the donor APC and DC depletion significantly reduced the number of the double positive cells in blood and lymph nodes of skin transplanted recipients (**Supplementary Figure 7C**), confirming the influence on recipient DC activity also by semi direct pathway. Interestingly, APC and cDC depletion in the donor more effectively targeted recipient DC activities and the subsequent Th cell mediated effector response to the skin grafts compared to the hind limb grafts (**Figures 2**, **4**, **5**). This implies that, despite disappearing earlier, donor DCs might play a role in the subsequent alloimmune response mediated by recipient DCs, albeit with organ-specific effects. Donor DCs can influence recipient DC activities in several different ways. After transmigrating to lymph nodes, the donor DCs may deliver cargo loaded with donor MHC and antigens to the recipient DCs, facilitating antigen presentation via indirect (cross-presentation) or semi direct (cross-dressing) pathways (43, 44). Additionally, donor DCs may secrete microvesicles-containing MHC-antigen complexes, which could be taken up by recipient DCs and presented to effector T cells via semi-direct mechanism. Depletion of APCs or DCs in the donor would likely interfere with all these ways of recipient DC activation.

Regardless of the transplantation model, Th17 cells exhibit reduced frequencies in the DC depletion groups, most prominently in skin transplant recipients (**Figure 5B**). Th17 cells play a crucial role in solid organ allograft rejection, and a decrease in their numbers has been associated with improved transplant outcomes (18, 45–48). It has been demonstrated that immunosuppression may not be sufficient to control Th17 mediated transplant rejection, as Th17 cells can exhibit variable resistance to immunosuppression (49–52). Additionally, a variable response to Treg mediated suppression was shown (53, 54). The reduced Th17 frequencies observed in our study could be a direct effect of depleted donor APCs or DCs, but it may also represent an indirect effect of the substantially reduced numbers of activated and matured recipient DCs. Since we rarely detected donor DCs in the analyzed WT secondary lymphoid organs at POD 6, the latter effect seems more likely to be responsible for reduction in Th17 cell counts. The decrease in Th17 cell counts in the APC and cDC depletion groups was also somewhat reflected by systemic and local cytokine expression in the allograft, particularly IL-17E and lymphotoxin-α (**Figure 5**). Although IL-17E is known to induce a Th2-mediated allergic response by stimulating IL-4, IL-5 and IL-13 production, several studies highlight its proinflammatory role in skin and autoimmune diseases (55). Genetic ablation or neutralization of IL-17E ameliorated skin inflammation induced by imiquimod and tape stripping by weakening innate immune responses (56). Contrarily, IL-17E has been shown to enhance the regulatory function of Foxp3 positive Tregs and prolong mouse skin graft survival (57). *In vivo* treatment of heart-transplanted rat recipients with lymphotoxin-α neutralizing antibodies prolonged graft survival (58). Likewise, the lymphotoxin-α KO mice showed a significant delay in the rejection of heart and skin grafts compared to the WT counterparts following splenectomy (59). Interestingly, in xenogeneic and allogeneic hematopoietic stem cell transplantation (hSCT) models, antibody mediated neutralization (60) or knockout of lymphotoxin-α but not of lymphotoxin-ß in the donor, led to the attenuation of graft versus host disease (GVHD) (61).

In summary, our findings suggest a higher sensitivity of the skin component in VCA compared to the underlying muscles and connective tissues. Correspondingly, the isolated skin graft demonstrated heightened immunogenicity compared to the whole VCA graft. Moreover, while depletion of donor APCs or cDCs was insufficient to ameliorate rejection of skin and hind allografts, the activities of recipient DCs and the subsequent Th17 response in the skin transplantation model were improved. Future studies exploring the effect of targeting donor DCs, along with immunosuppression, may offer additional benefits and warrant further investigation. Additionally, future studies should aim to understand the precise mechanisms by which donor DC regulate the activity of the recipient DCs, conducting a thorough analysis of the temporal dynamics of donor DCs and their interaction with the recipient DCs. The inevitable IRI of the transplant results in the release of damage-associated molecular patterns (DAMPs) from the injured cells, which promote activation of APCs, including DCs. Future studies should explore how the IRI of skin and hind limb grafts influence the activation and migration of graft-resident DCs to the lymphoid organs of the recipient and impacts graft outcome.

## Data availability statement

The raw data supporting the conclusions of this article will be made available by the authors, without undue reservation.

## Ethics statement

The animal study was approved by The State Office for Health and Social Affairs (Landesamt für Gesundheit und Soziales), Berlin (approval number: G0300/17). The study was conducted in accordance with the local legislation and institutional requirements.

## Author contributions

MA: Conceptualization, Data curation, Formal analysis, Investigation, Methodology, Writing – original draft, Writing – review & editing. JM: Data curation, Formal analysis, Investigation, Methodology, Writing – review & editing. AR-S: Data curation, Formal analysis, Investigation, Validation, Writing – review & editing. DP: Data curation, Methodology, Writing – review & editing, Validation. KF: Data curation, Methodology, Writing – review & editing. SL: Data curation, Methodology, Writing – review & editing. PT: Data curation, Methodology, Writing – review & editing. EM: Data curation, Formal analysis, Methodology, Writing – review & editing. RC: Data curation, Formal analysis, Writing – review & editing. JP: Conceptualization, Funding acquisition, Resources, Supervision, Writing – review & editing. CW: Investigation, Resources, Supervision, Writing – review & editing. IS: Project administration, Resources, Supervision, Validation, Writing – review & editing. ST: Funding acquisition, Project administration, Supervision, Validation, Writing – review & editing. BK: Data curation, Formal analysis, Investigation, Methodology, Project administration, Writing – original draft, Writing – review & editing.
