# Supplementary material for: Depletion of donor dendritic cells ameliorates immunogenicity of both skin and hind limb transplants
Source: Front Immunol. 2024 May 10;15:1395945. doi: 10.3389/fimmu.2024.1395945 (PMC11116604; doi:10.3389/fimmu.2024.1395945)
Supplement: Supplementary file 1 [file DataSheet_1.docx]

**Supplementary Information**

**Supplementary Figure 1: Clodronate treatment in C57Bl/6 mice and DT treatment to zDC-DTR mice effectively decrease numbers of APCs and DCs, respectively.** Immune cells were isolated from spleen and blood of the untreated (WT) C57Bl/6J mice, DT treated zDC-DTR mice and clodronate treated C57Bl/6J mice and analyzed by flow cytometry to assess abundance of DCs (CD11b^-^CD11c^+^) **(A)** and macrophages (F4/80^+^) **(B)**. (C) Immune cells were isolated from skin and hind limb grafts of the untreated (WT), APC depleted (CL; clodronate treated) and cDC depleted (zDC; DT treated zDC-DTR) groups at POD 6 and analyzed for the abundance of donor specific DCs by flow cytometry. The bar graphs indicate frequencies of donor specific DCs (CD11c^+^H2Kb^+^). All values are given in % of the described population on the ordinate/”y-axis”. (n=8 skin Tx, n=5 hind limb Tx). *p<0.05, ***p<0.001.

**Supplementary Figure 2: Gating strategy for identification of T cell subsets and NK cells.** Recipient specific live immune cells were defined after gating on live H-2Kb^-^ cells and further classified into subsets of T lymphocytes and NK using specific markers, as indicated by black arrows. FSC: forward scatter, SSC: side scatter.

**Supplementary Figure 3: Gating strategy for identification of dendritic cell subsets.** Recipient specific live immune cells were defined after gating on live H-2Kb^-^ cells and further classified into several subsets of dendritic cells using specific markers, as indicated by black arrows. FSC: forward scatter, SSC: side scatter, DCs: dendritic cells.

**Supplementary Figure 4: Gating strategy for identification of Treg and Th17 cells.** Recipient specific live immune cells were defined after gating on live H-2Kb^-^ cells and further classified into Treg and Th17 cells using specific markers, as indicated by black arrows. FSC: forward scatter, SSC: side scatter, DCs: dendritic cells.

**Supplementary Figure 5: Effect of donor APC and cDC depletion on the systemic levels of proinflammatory and regulatory cytokines.** Serum was isolated from the blood samples of skin and hind limb transplanted mice at POD 6 and analyzed for cytokine levels using multiplex immunoassay. The representative bar graphs indicate their relative systemic levels in recipients of untreated (WT) C57Bl/6J mice, clodronate treated (CL: APC depleted) C57Bl/6J mice and DT treated (zDC: cDC depleted) zDC-DTR mice. (n= 3). *p<0.05

**Supplementary Figure 6: Effect of donor APC and cDC depletion on intragraft expression of proinflammatory cytokines.** Quantitative RT-PCR analysis of IFN-γ, TNF-α, IL-6 and IL-17A in the skin and hind limb grafts at POD 6. (n= 4). *p<0.05

**Supplementary Figure 7: (A, B) Gating strategy for identification of donor and recipient specific DCs. (A)** DCs were defined by its lineage specific marker (CD11c^+^) after gating on live CD11b^-^ cells. DCs were then further sub-classified into donor and recipient DCs by using H2Kb and H2Kd markers, respectively. **(B)** Recipient DCs and double positive DCs were further classified based on the maturation (MHCII^+^) and activation (CD40^+^) phenotypes. **(C) Effect of APC and cDC depletion on DCs, expressing both donor and recipient specific MHC-I molecules.** Immune cells were isolated from blood and lymphoid tissues (spleen and lymph nodes) of the recipients (DBA/2 mice) of skin and hind limb grafts derived from untreated (WT), APC depleted (CL; clodronate treated) and cDC depleted (zDC; DT treated zDC-DTR) donor mice at POD 6 and analyzed by flow cytometry. The bar graphs indicate frequencies of cross-dressed double positive (H2Kb+H2Kd+) DCs. All values are given in % of the described population on the ordinate/”y-axis”. (n=8 skin Tx, n=5 hind limb Tx). *p<0.05, **p<0.01.

**Supplementary Figure 8: Gating strategy for identification of donor specific lymphocytes in the recipient lymphoid tissues and peripheral blood.** Donor derived immune cells were defined by their expression of donor specific marker (H2Kb) and absence of recipient specific marker (H2Kd) after gating on live single cells. FSC: forward scatter, SSC: side scatter. **Tables**

**Supplementary Table 1**: Antibodies used for immune cell phenotyping

| **Antigen** | **Fluorochrome** | **dilution** | **Cat. #** | **Company** |
| --- | --- | --- | --- | --- |
| CD3 | PE | 1:200 | 100308 | BioLegend |
| CD4 | PE-Cy7 | 1:800 | 25-0042-82 | Thermo Scientific |
| CD8 | APC | 1:200 | 100712 | BioLegend |
| CD11b | APC-Cy7 | 1:400 | 101226 | BioLegend |
| CD11c | PE-Cy7, PE | 1:400 | 117318, 117308 | BioLegend |
| CD16/32 | APC | 1:200 | 101326 | BioLegend |
| CD25 | PerCP-Cy5.5 | 1:200 | 102030 | BioLegend |
| CD45R/B220 | PerCP-Cy5.5 | 1:200 | 103236 | BioLegend |
| CD45 | PE-TexasRed | 1:400 | 103146 | BioLegend |
| CD40 | FITC | 1:100 | 102910 | BioLegend |
| CD62L | APC-Cy7 | 1:400 | 104428 | BioLegend |
| CD80 | APC | 1:200 | 104714 | BioLegend |
| H-2Kb | FITC, PE | 1:100, 1:200 | 116510, 116508 | BioLegend |
| H-2Kd | FITC | 1:100 | 116608 | BioLegend |
| Helios | APC | 1:30 | 137222 | BioLegend |
| I-A/I-E (MHCII) | PerCP-Cy5.5 | 1:200 | 107626 | BioLegend |
| IL-17A | FITC | 1:100 | 11-7177-81 | Thermo Scientific |
| NKp46 | FITC | 1:100 | 137606 | BioLegend |
|  |  |  |  |  |

**Supplementary Table 2:** qPCR probes/primer? used for cytokine mRNA expression analysis

| **Cytokine** | **Assay ID** | **Cat #** | **Company** |
| --- | --- | --- | --- |
| IL-6 | Mm00446190_m1 | 4331182 | Thermo Scientific |
| IL-17A | Mm00439618_m1 | 4331182 | Thermo Scientific |
| IL-17E/IL-25 | Mm00499822_m1 | 4331182 | Thermo Scientific |
| IFNγ | Mm01168134_m1 | 4331182 | Thermo Scientific |
| TNF-α | Mm00443258_m1 | 4331182 | Thermo Scientific |
| TNF-β/LT-α | Mm00440228_gH | 4331182 | Thermo Scientific |
| Ppia | Mm02342430_g1 | 4331182 | Thermo Scientific |
