# Supplementary figures and images for: Depletion of donor dendritic cells ameliorates immunogenicity of both skin and hind limb transplants
Source: Front Immunol. 2024 May 10;15:1395945. doi: 10.3389/fimmu.2024.1395945 (PMC11116604; doi:10.3389/fimmu.2024.1395945)

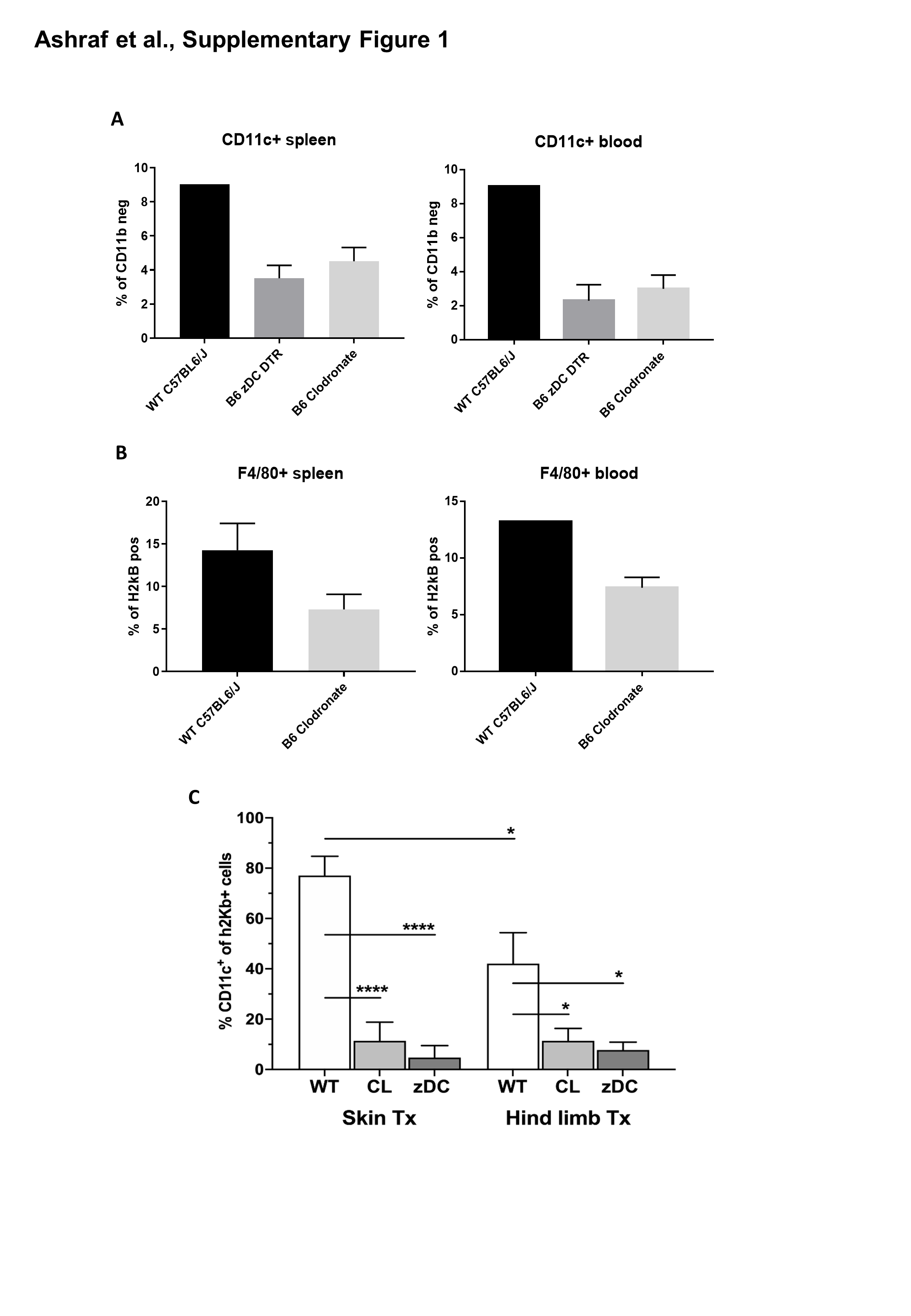

Supplement: Supplementary file 2 [file Image_1.tif]

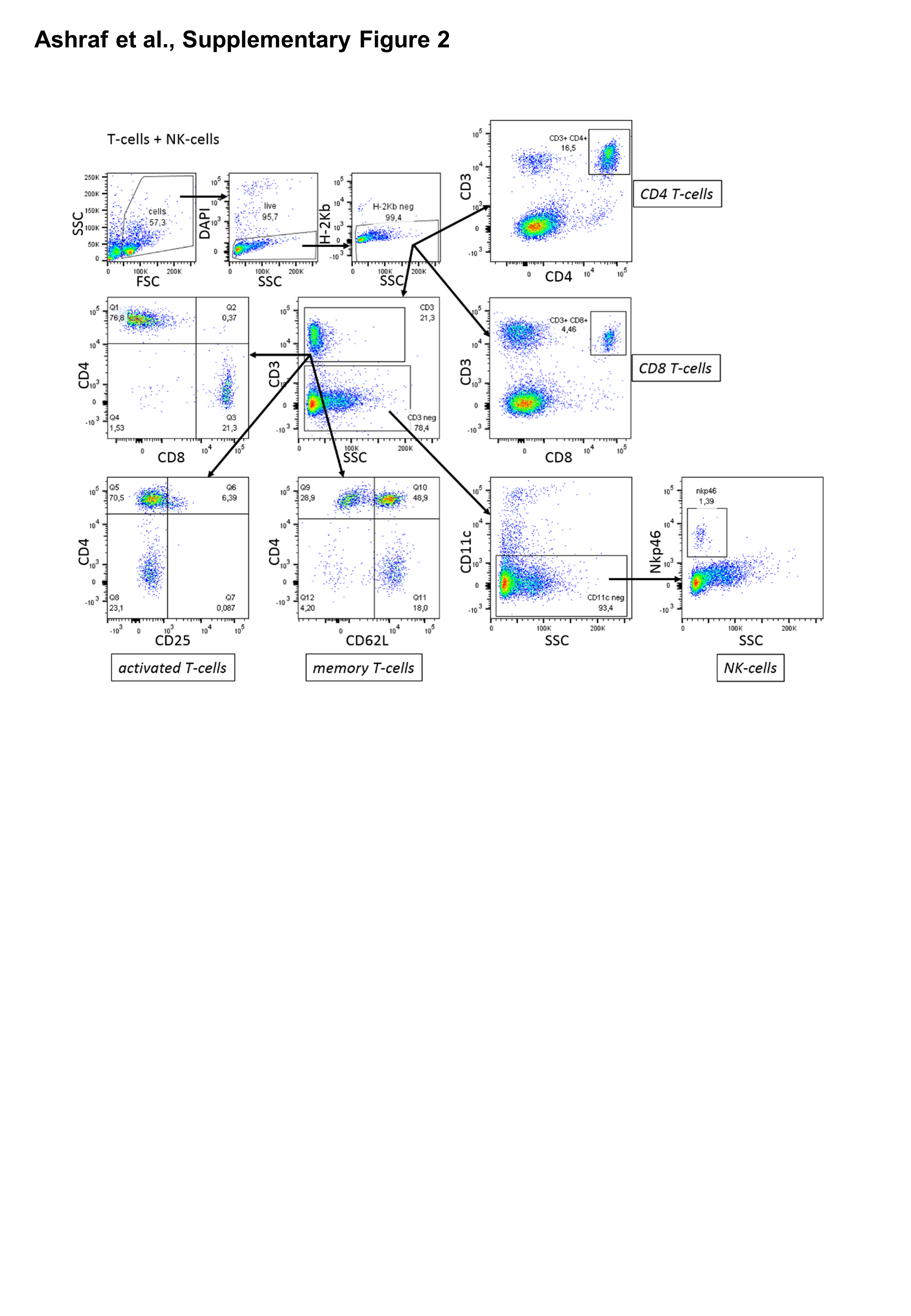

Supplement: Supplementary file 3 [file Image_2.tif]

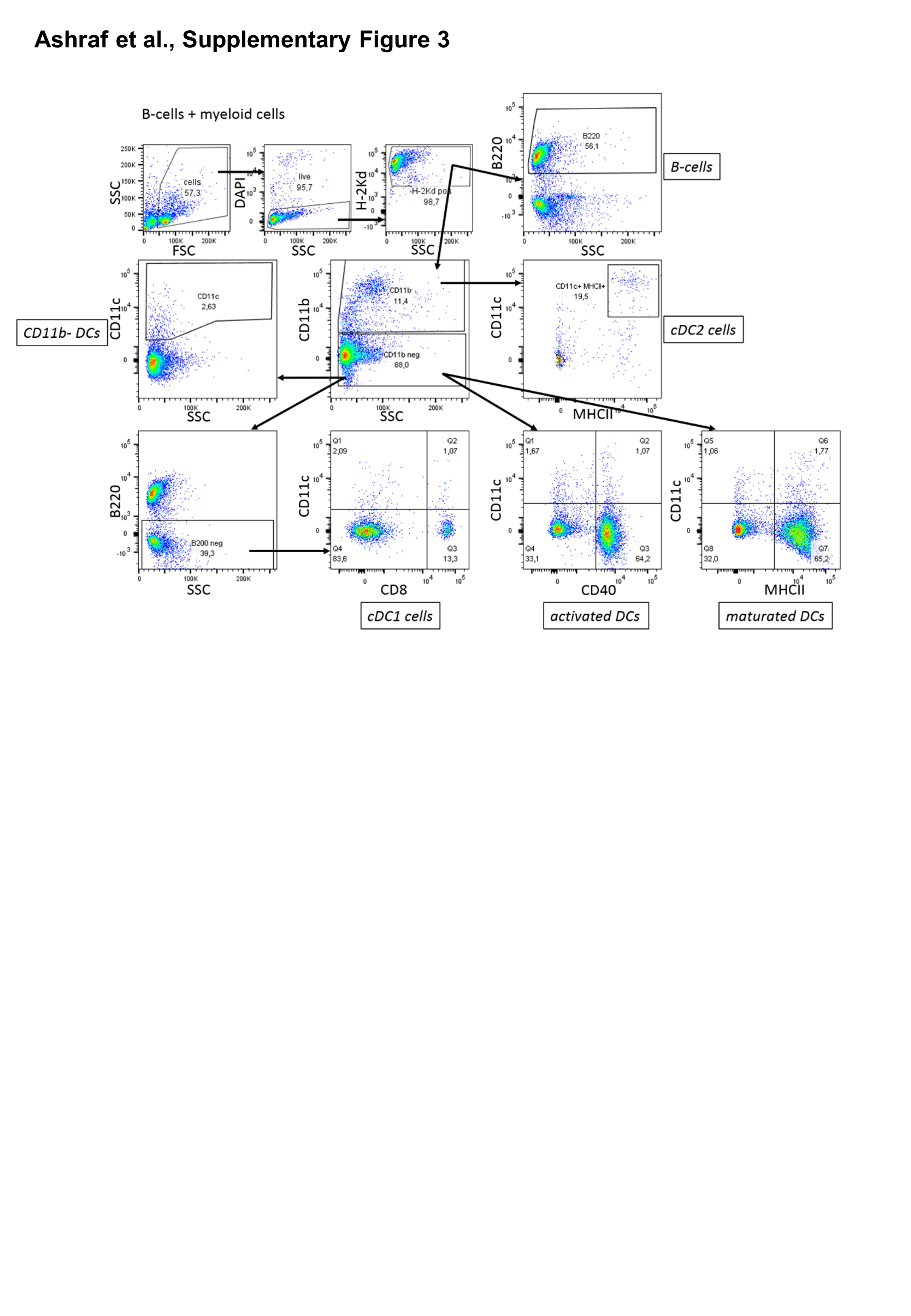

Supplement: Supplementary file 4 [file Image_3.tif]

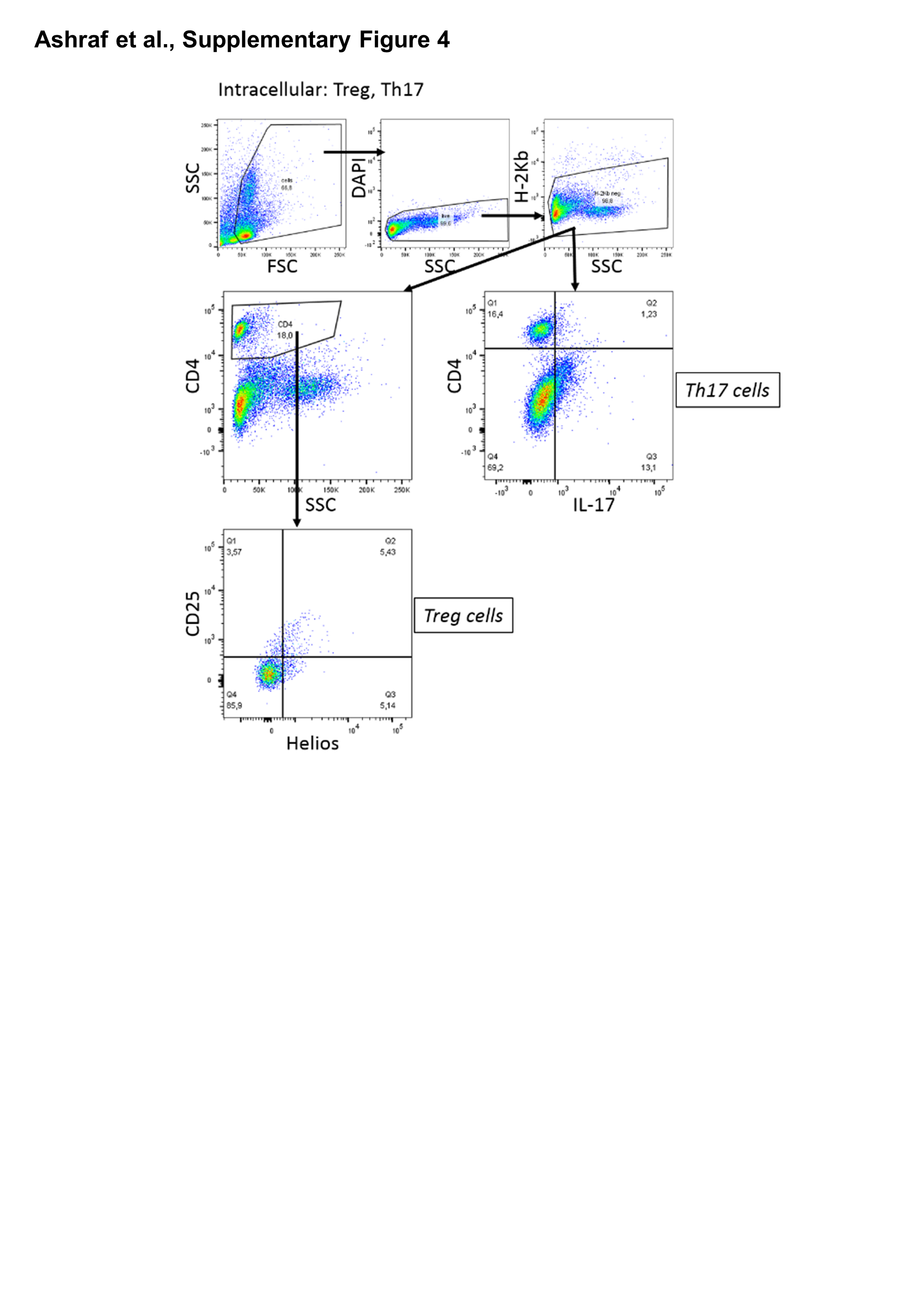

Supplement: Supplementary file 5 [file Image_4.tif]

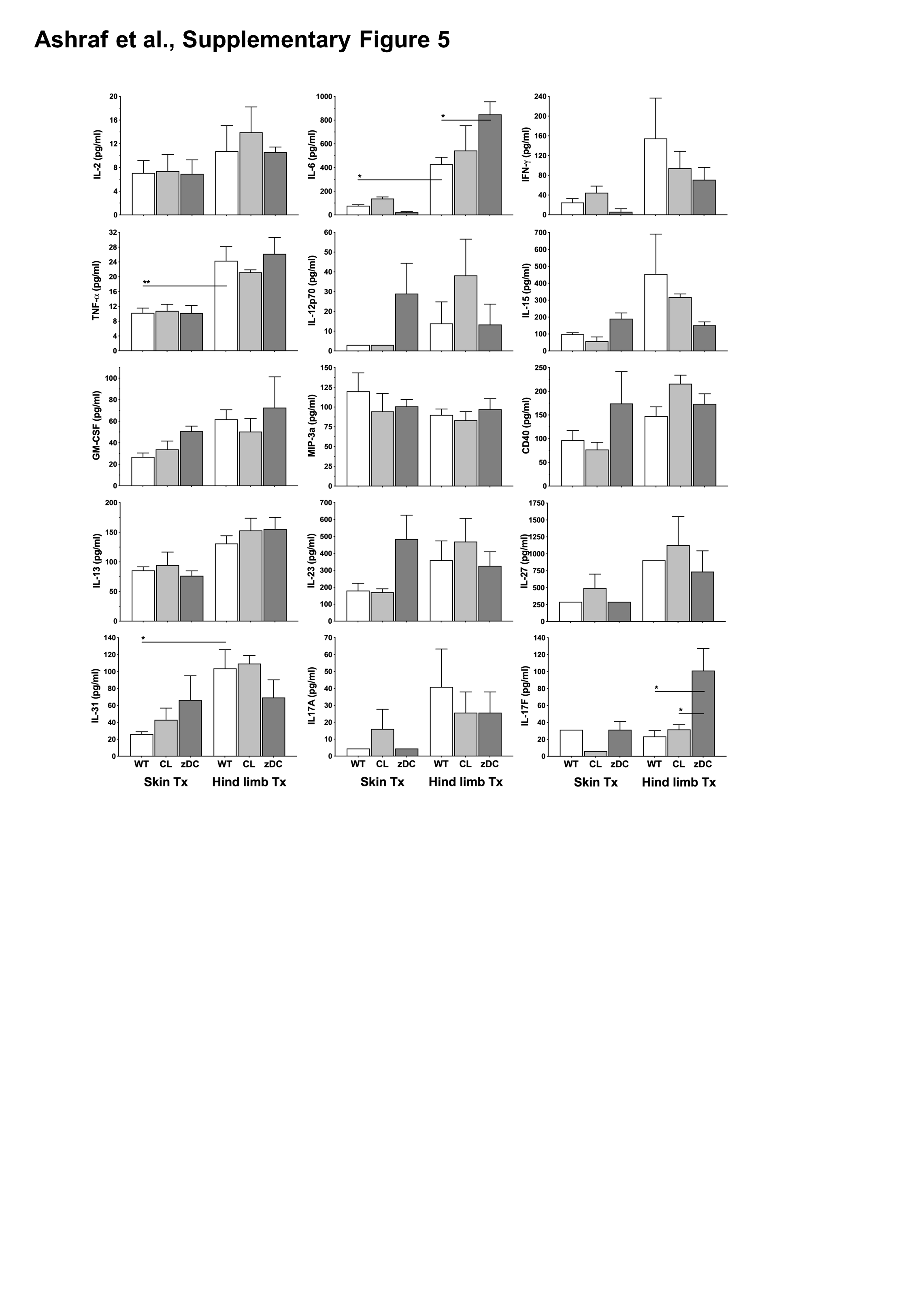

Supplement: Supplementary file 6 [file Image_5.tif]

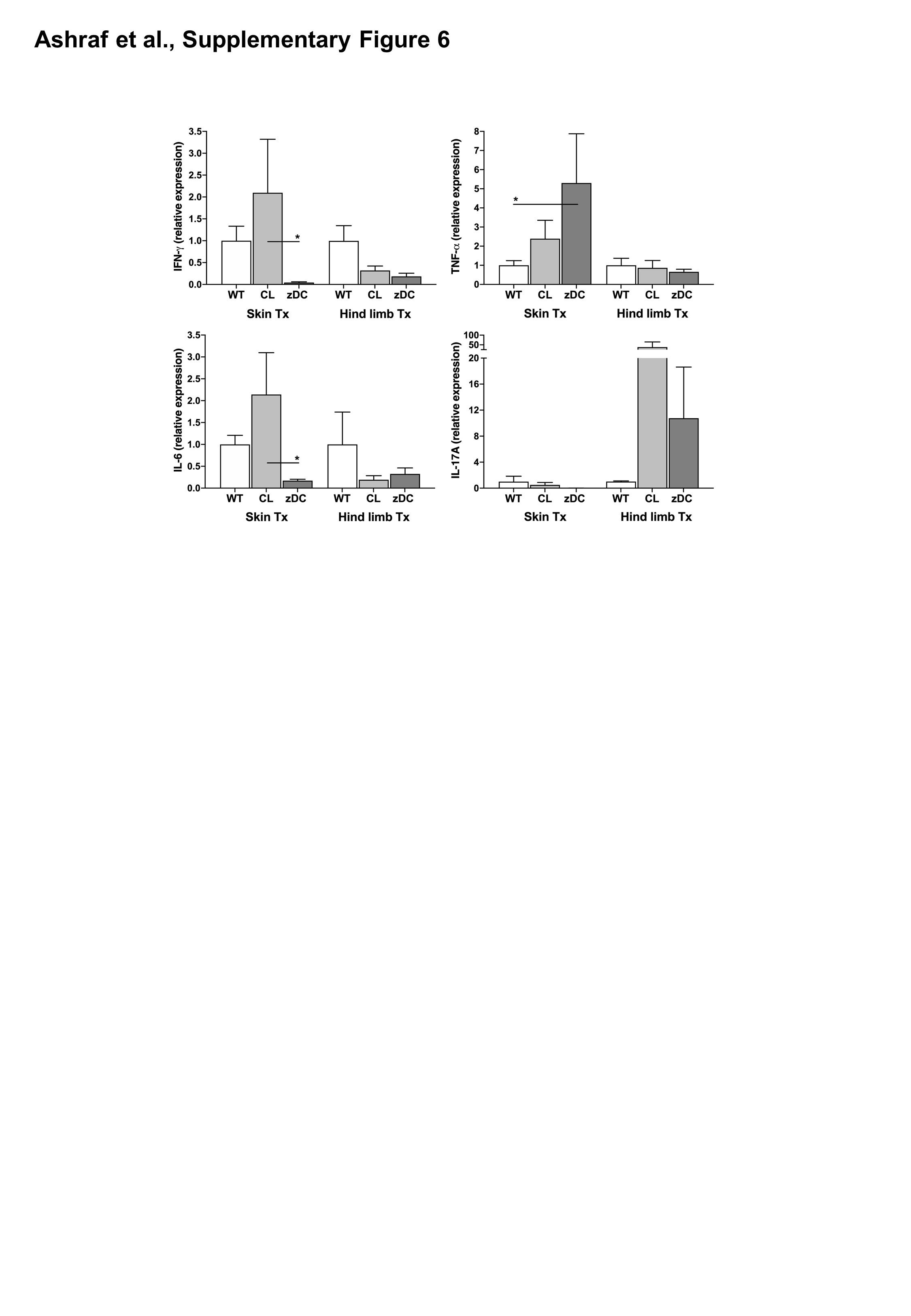

Supplement: Supplementary file 7 [file Image_6.tif]

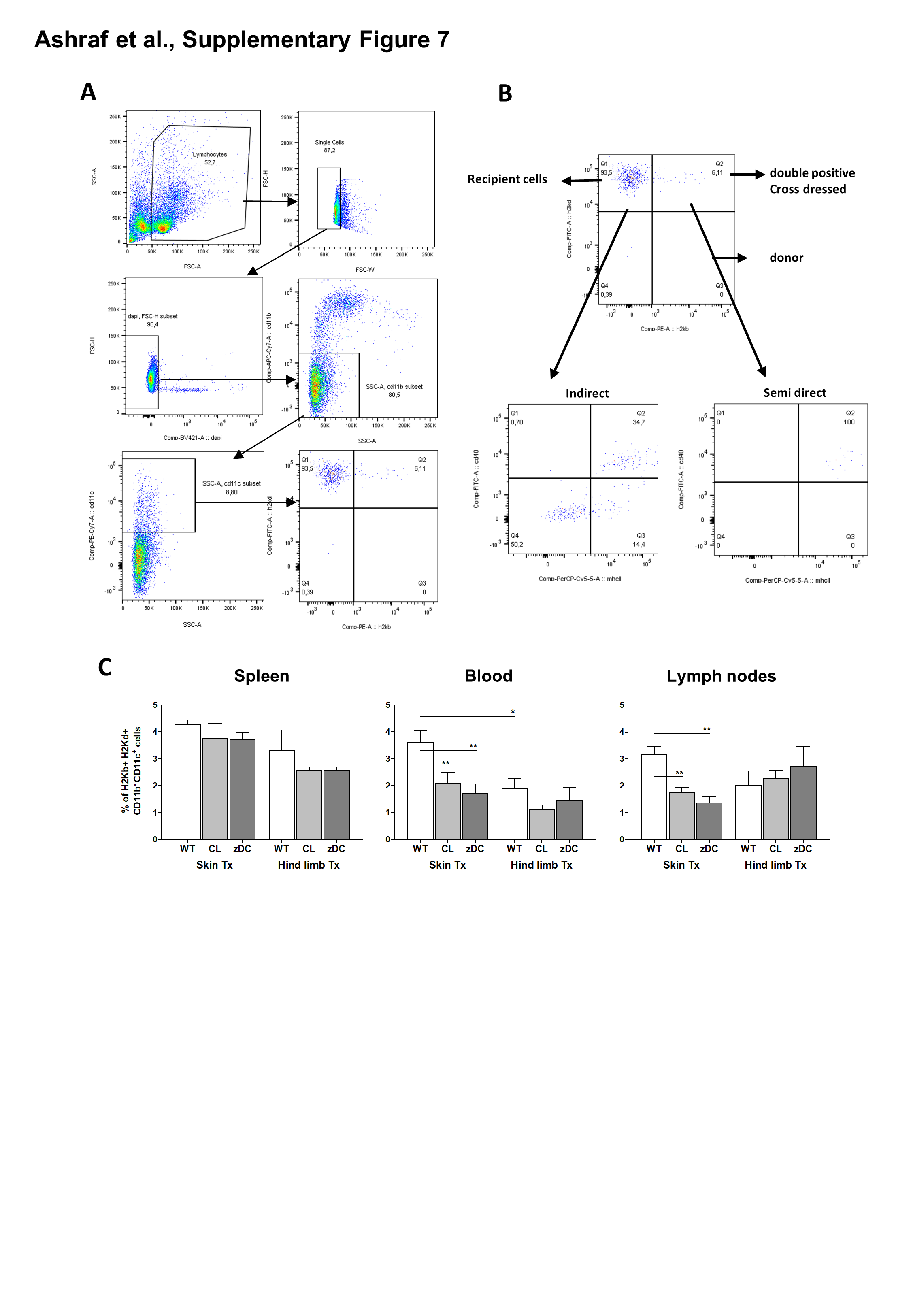

Supplement: Supplementary file 8 [file Image_7.tif]

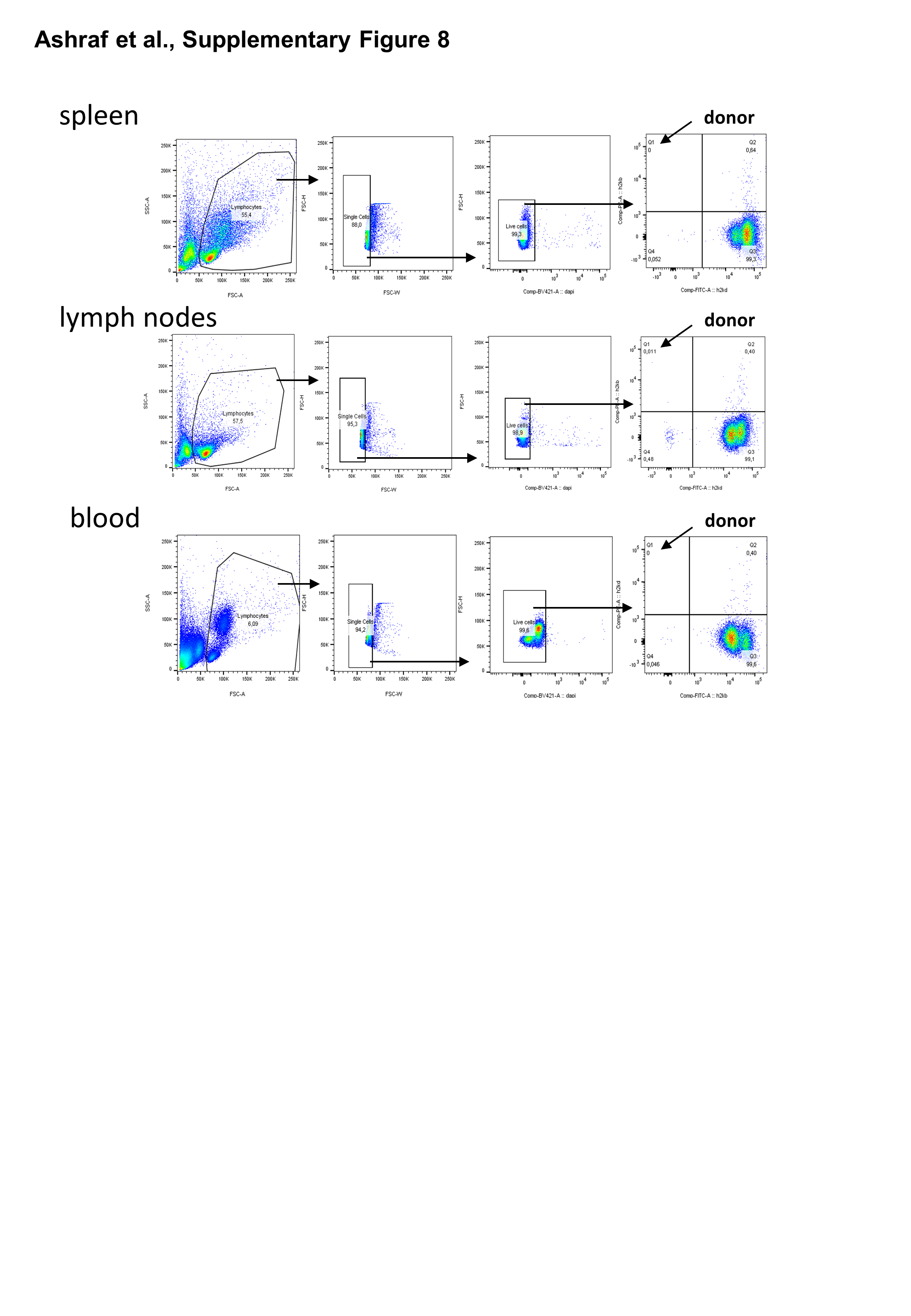

Supplement: Supplementary file 9 [file Image_8.tif]
